# Supplementary material for: Postoperative Observation of Spaying with the Silicon Ring on the Ovaries in Heifers: A Retrospective Study in 28 Cases
Source: Vet Sci. 2022 Nov 21;9(11):643. doi: 10.3390/vetsci9110643 (PMC9696694; doi:10.3390/vetsci9110643)
Supplement: Supplementary file 1 [file vetsci-09-00643-s001.zip › vetsci-1966148-supplementary.pdf]

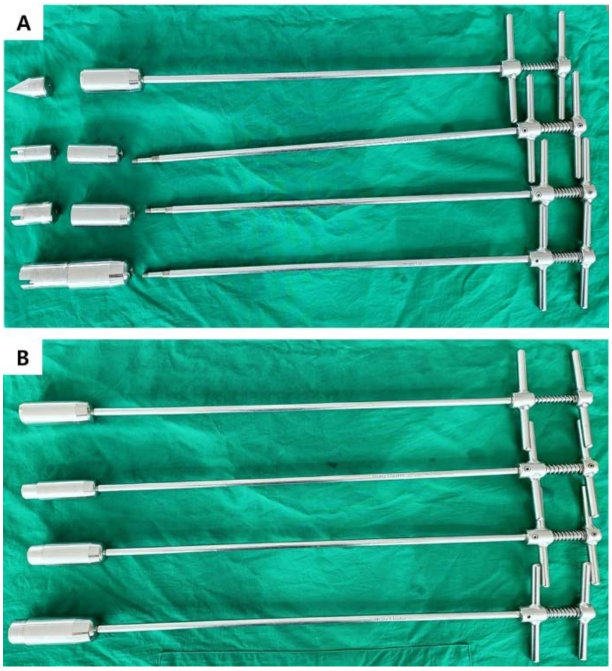

Supplementary Figure S1. The instruments (perforator and ligators) for spaying in cattle. (A) Unassembled parts of stainless-steel instrument. The T bar shafts are about 45 cm long and the sockets are about 5-10 mm inner diameter; the values vary for different age and size of animals. The bars can be assembled with a spear head or socket for a perforator or ligator, respectively. (B) Assembled perforator (top) and ligators. At the end of the socket in the ligators, the silicon ring can be fitted at the outer margin.
